# Sizing Gaseous Emboli Using Doppler Embolic Signal Intensity

**DOI:** 10.1016/j.ultrasmedbio.2012.01.008

**Published:** 2012-05

**Authors:** Caroline Banahan, James P. Hague, David H. Evans, Rizwan Patel, Kumar V. Ramnarine, Emma M.L. Chung

**Affiliations:** ∗ Medical Physics Department, University Hospitals of Leicester NHS Trust, Leicester, United Kingdom; † Department of Physical Sciences, The Open University, Milton Keynes, United Kingdom; ‡ Department of Cardiovascular Sciences, University of Leicester, Leicester, United Kingdom

**Keywords:** Embolus detection, Transcranial Doppler ultrasound, Gaseous emboli

## Abstract

Extension of transcranial Doppler embolus detection to estimation of bubble size has historically been hindered by difficulties in applying scattering theory to the interpretation of clinical data. This article presents a simplified approach to the sizing of air emboli based on analysis of Doppler embolic signal intensity, by using an approximation to the full scattering theory that can be solved to estimate embolus size. Tests using simulated emboli show that our algorithm is theoretically capable of sizing 90% of “emboli” to within 10% of their true radius. *In vitro* tests show that 69% of emboli can be sized to within 20% of their true value under ideal conditions, which reduces to 30% of emboli if the beam and vessel are severely misaligned. Our results demonstrate that estimation of bubble size during clinical monitoring could be used to distinguish benign microbubbles from potentially harmful macrobubbles during intraoperative clinical monitoring.

## Introduction

Transcranial Doppler (TCD) detection of high numbers of air bubbles entering the cerebral circulation during cardiac surgery and suspected links between air embolism and cognitive decline, provide a strong clinical motivation for extending ultrasound embolus detection techniques to the prediction of bubble size. Approximately a third of patients undergoing cardiac surgery are found to emerge with postsurgical neurocognitive decline (Newman et al. 2001). However, studies designed to identify a link between embolisation and poor postoperative outcome have so far proved inconclusive (Martin et al. 2009a; Jacobs et al. 1998). One reason for this may be that TCD is sensitive to the tiniest of air bubbles but is currently not used to distinguish between benign fast-dissolving microbubbles and clinically significant air emboli. The capacity to estimate bubble size during intraoperative monitoring would prove useful in understanding the clinical significance of air emboli and in improving our understanding of brain injury following cardiac surgery (Chung et al. 2007).

In previous attempts to size bubbles using ultrasound Lynch et al. (2007) attempted to size gaseous microemboli in extracorporeal blood circuits using an analytical model based on scattering theory (Anderson 1950). Their model was validated experimentally for bubble diameters ranging between 50 and 130 μm and they concluded that accurate size estimation was possible using the backscattered signal provided that the spacing of emboli was sufficient to minimise interference and other multi-path scattering effects, but they did not establish an algorithm for inverting that theory to predict embolus sizes. Palanchon et al. (2003b) utilised the nonlinear behaviour of bubbles to size gaseous microemboli by studying the subharmonic and ultraharmonic emissions from small bubbles with diameters ranging between 40 and 120 μm. However, the range of embolus diameters that can be sized using this technique is limited to small bubbles due to the finite range of frequencies generated by the transducer.

The scattering theory model used as the basis for embolus sizing described in this article was first proposed by Moehring and Klepper (1994) who extended a previous model by Lubbers and van den Berg’s (1977) of backscattering of ultrasound from a spherical embolus in a blood-filled vessel to incorporate red-cell aggregation effects using a derived blood backscatter coefficient (Mo 1990). Lubbers and van den Berg’s original expression was derived from standard acoustic scattering theory, see for example Rzhevkin (1963), and Morse and Ingard (1968). Equation (1) gives the expression used by Moehring and Klepper (1994) for calculating backscattered ultrasound intensity in the form of an embolus to background ratio (EBR). The EBR describes the ratio of the backscatter from flowing blood with and without an embolus present, defined as 10 log_10_(σ_EMBOLUS_/σ_RBC_), where σ_RBC_(*f*) is the backscatter cross-section for red blood cells at frequency *f* (surrounded by plasma) and σ_EMBOLUS_(*f*) the embolus cross-section at frequency *f*. A ratio is used as this simplifies handling of variations in the power and geometry of the beam. The full scattering expression relating an embolus with radius *r_0_* to EBR is complicated to invert to obtain the embolus size. Expressed in logarithmic form:

(1) $$\begin{array}{c} \begin{array}{c} 10\;\log_{10}\left(EBR\right)=10\;\log_{10}\left({\left(\frac{c}{2\pi f}\right)}^{2}{\left|\sum_{m=0}^{\infty }{\left(-1\right)}^{m}a_{m}P_{m}\left(\cos\vartheta \right)i^{m+1}\right|}^{2}\right)\\ -20\;\log_{10}\left(\pi R\right)\\ -10\;\log_{10}\left(\frac{H{\left(1-H\right)}^{4}}{H_{0}{\left(1+2H\right)}^{2}}\right)\\ -10\;\log_{10}\left(\sigma_{RBC}\left(f\right)\right)\\ -10\;\log_{10}\left(\delta \right)\\ -10\;\log_{10}\left(L\right) \end{array} \end{array}\left(\text{dB}\right)$$

where

$$a_{m}=-i^{m}\left(2m+1\right)\frac{\bar{\rho }z_{0}j_{m+1}{\bar{j}}_{m}-\rho {\bar{z}}_{0}j_{m}{\bar{j}}_{m+1}+\left(\rho -\bar{\rho }\right)mj_{m}{\bar{j}}_{m}}{\bar{\rho }z_{0}h_{m+1}{\bar{j}}_{m}-\rho {\bar{z}}_{0}h_{m}{\bar{j}}_{m+1}+\left(\rho -\bar{\rho }\right)mh_{m}{\bar{j}}_{m}}$$

Here, *f* is the carrier frequency, $z_{0}=2\pi fr_{0}/c$, ${\bar{z}}_{0}=2\pi fr_{0}/\bar{c}$, where *ρ* and *c* are the density and speed of sound of whole blood, and $\bar{\rho }$ and$\;\bar{c}$ correspond to the density and speed of sound of the embolus. The functions $j_{m},{\bar{j}}_{m}$ are spherical Bessel functions of order *m* (having arguments of *z*_0_ and ${\bar{z}}_{0}$, respectively), *h_m_* is a spherical Hankel function of the second kind of order *m* and having argument *z*_0_, *P_m_(cosϑ)* is a Legendre polynomial of order *m*, $W={\left(1-H\right)}^{4}/{\left(1+2H\right)}^{2}$ where *W* is the packing factor for an elemental acoustic volume or voxel and H is the percentage haematocrit (the ratio of blood volume with plasma removed to total blood volume). R is the vessel radius, L is the sample volume length (SVL) and δ = 5 × 10^9^ cm^−3^ is the number density of red cells at 45% haematocrit (H_0_) (Atkinson and Woodcock 1982).

The complexity of these equations has previously made them difficult to apply to the interpretation of embolic signals in a clinical setting. In this study, we investigate whether sizing of bubbles can be achieved by comparing the intensity of Doppler embolic signals to predictions based on scattering theory via an approximation. Scattering theory is capable of predicting the backscattered ultrasound signal intensity from gaseous emboli of different sizes travelling in a blood-filled vessel (Lubbers and van den Berg 1977) but is mathematically complicated. To make sizing of bubbles more accessible to clinical researchers, we have developed and tested an algorithm for conversion of backscattered ultrasound intensities to bubble size based on an approximation to the full scattering theory which can be inverted by the straightforward solution of a cubic equation. Our expression is first tested against exact results from the full scattering theory by sizing distributions of 10,000 simulated “emboli” to identify deviations from the full scattering theory that are intrinsic to the algorithm. A laboratory validation study investigating the impact of measurement uncertainty and beam misalignment is also presented to evaluate whether our algorithm would be capable of predicting bubble sizes *in vivo*.

## Methods

### Sizing algorithm

**Fig. 1:**
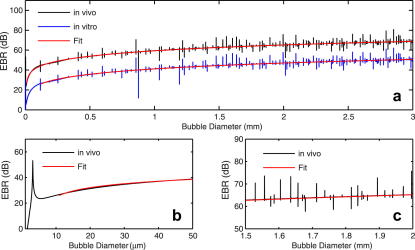
(a) Theoretical relationship between embolus to blood ratio (EBR) values and embolus size for spherical air bubbles *in vivo* and *in vitro*. The red line shows the approximated fitting function for both. The vessel diameter was 4 mm in the *in vitro* calculation to match our experiment and 2.5 mm was assumed as the diameter of the middle cerebral artery (MCA) *in vivo*. (b) The characteristic resonance peak for 3 μm bubbles insonated with 2 MHz ultrasound. (c) Resonant structures along the scattering curve are due to spherical harmonics from modelling the scattering of a plane wave from a spherical bubble and observing the backscatter at exactly 180°.

**Fig. 2:**
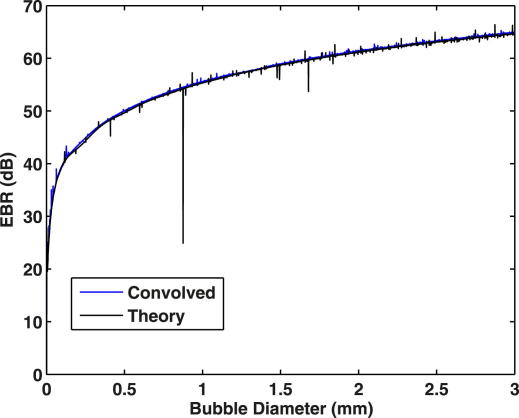
The theoretical scattering curve for bubbles 1 μm to 3 mm in diameter insonated with a 2 MHz transducer compared with the same curve convolved with the fast Fourier transform of a 16-cycle, 2 MHz carrier frequency sine wave of 8 μs duration. The sharp structures on the theoretical curve due to higher spherical harmonics are not as pronounced when the finite bandwidth of the transducer is taken into consideration.

The aim in developing a bubble sizing algorithm was to be able to input relevant parameters such as EBR, vessel diameter, sample volume length and haematocrit and output an estimated embolus size and associated uncertainty in that value. Numerical values used for modelling the backscatter of air emboli in blood *in vivo* and for specific parameters associated with our *in vitro* laboratory set-up are summarised in Table 1. For 2 MHz ultrasound, the scattering theory predicts the theoretical relationship between EBR and bubble size shown in Figure 1. A sharp resonance is observed for ∼3 μm diameter bubbles (Fig. 1b) associated with the characteristic resonance frequency of air bubbles insonated at 2 MHz. For diameters above 3 μm the model predicts a near monotonic relationship between backscattered signal intensity and diameter. This considerably simplifies the problem of sizing of emboli as each measured EBR (MEBR) corresponds to a unique solution for embolus size. Although very narrow resonant structures can also be observed along the curve due to higher spherical harmonics (Fig. 1c), it is not anticipated that these sharp structures would be discernible experimentally due to the ultrasound system’s finite bandwidth. To determine how the finite bandwidth of the transducer would affect our ability to see these resonances, we calculated the backscattered cross-section for individual bubbles (diameters ranging between 1 μm and 3 mm) for insonating frequencies between 0.5 and 32 MHz in 0.5 MHz steps and convolved this frequency spectrum for each individual bubble with the frequency response of our transducer (16-cycle, 2 MHz pulse of 8 μs duration). The weighted backscattered cross-sections were then summed and the theoretical scattering curve recalculated. Figure 2 displays the resulting scattering curve and the resonant structures are now barely noticeable.

To approximate the full scattering curve while excluding resonance effects, we fit a simple expression to the theory based on a Padé approximant [eqn (2)]. The Padé approximant preserves the general monotonic relationship between EBR and embolus size while neglecting sharp resonances:

(2) $$EBR=m\left(\frac{x^{2}+bx+c}{x^{2}+dx+e}\right)x+g$$

The variable *x* is log_10_(*r/r_0_)*, where *r* is the embolus radius in metres, r_0_ is 1 m and the curve coefficients (*m* = 977.5, *b* = 17.24, *c* = 67.80, *d* = 285.45, *e* = 1663.71 and g = 155.125) were identified by nonlinear least squares regression in Matlab (nlinfit, Mathworks Inc., Natick, MA, USA). Fitting was performed between 10 μm and 3 mm to exclude the main resonance at 3 μm. Figure 1 shows the fitted function for backscattering of 2 MHz ultrasound from spherical air emboli in a blood-filled 2.5 mm diameter vessel, and for our *in vitro* experiment, as the red solid line.

By making use of the logarithmic form of eqn (1), the coefficient, g, in eqn (2) may be altered to incorporate differences in vessel radius (R) and haematocrit (H) by introducing the following terms:

(3) $$g\prime =g-20\;\log_{10}\left(\frac{R}{R_{0}}\right)-10\;\log_{10}\left(\frac{\delta H}{H_{0}}\right)$$

R_0_, δ and H_0_ are the parameters used in the scattering theory curve which the Padé Approximant [eqn (2)] was fitted to. Any new value for vessel radius and haematocrit input into g′ will scale the fitted curve accordingly. Thus, it is possible to scale the EBR vs. embolus diameter curve for individual patients to provide a more accurate estimation of bubble size.

Using the above coefficients and eqn (3), eqn (2) can be reduced to a cubic function (see Abramowitz and Stegun 1964) [eqn (4)] to estimate embolus diameters up to 3 mm, which is approximately the diameter of the middle cerebral artery through which the emboli are travelling. This corresponds to EBR values of up to 70 dB:

(4) $$mx^{3}+\left(mb+g\prime -EBR\right)x^{2}+\left(mc+g\prime d-EBRd\right)x+g\prime e-eEBR=0$$

For every EBR input into eqn (4), a cubic function is produced which when solved will produce three roots, *x_*1*_*, *x_*2*_* and *x_*3*_*. In this study, the equation was solved for *x* using the roots function in Matlab (version 7.8.0 [R2009a]). Of the three roots returned, x_3_ was the real root (*x_r_*), with the other two roots being complex. The fit to the theory was made using this real root, *x_r_*_,_ where the radius of the embolus is given by eqn (5):

(5) $$r=r_{0}10^{x_{r}}$$

To allow sizing of emboli with embolic signals from 0 dB up to 70 dB the cubic function was tested to ensure that only one of the roots, *x_r_* was real and continuous for all EBR values in this range. Although this method for estimating embolus size involves solving a cubic equation, it is considerably simpler than performing inversion of eqn (1), which can also lead to values that are not unique. It would be easy to generate look-up tables with this simplified approach where different parameter values could be considered, *e.g*., vessel diameter or haematocrit, and bubbles could be sized according to their EBR values for the closest matching set of parameters.

To test our inversion function and evaluate the impact of neglecting resonant structures on the scattering curve, 10,000 simulated “air bubbles” with diameters from 1 μm up to 3.5 mm were randomly generated with two probability distributions; a flat distribution to determine whether size estimates were adversely affected at particular diameters and a more clinically realistic power law distribution reflecting the higher prevalence of small microbubbles compared with large macrobubbles (Barak and Katz 2005; Lynch et al. 2007). EBR values for specific radii of emboli were calculated using the full scattering theory [eqn (1)] and then input into our simplified function [eqns (4) and (5)], to make predictions for embolus size. By comparing the original distribution with our algorithm predictions, it was possible to test for inaccuracies introduced purely as a consequence of our algorithm.

### *In vitro* flow-rig

**Fig. 3:**
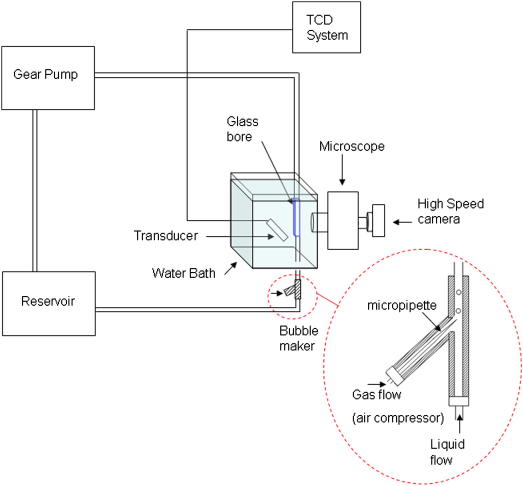
Diagram of the *in vitro* experiment used to measure peak embolic signal intensity corresponding to various sized air bubbles. The flow circuit was filled with a blood mimicking fluid (BMF) and nonpulsatile flow between 25 and 30 cms^−1^ was generated using a programmable gear pump.

To test whether our algorithm could predict bubble sizes *in vitro,* we developed a flow phantom to generate and image bubbles of known size. A schematic of this phantom is provided in Figure 3. A programmable gear pump (Micropump Model 120-000-1100; Micropump Corporation, Concord, CA, USA) was used to generate controllable steady flow of a diluted blood mimicking fluid (BMF) (Ramnarine et al. 1998). The circuit was constructed from C-flex tubing with 4 mm internal diameter and 0.8 mm wall thickness (Cole-Parmer, London, UK). C-flex has a density of 886 kgm^−3^, acoustic velocity of 1,556 ms^−1^ and attenuation of 5 dBcm^−1^MHz^−1^, compared with 1000 kgm^−3^, 1540 ms^−1^ and 0.5 dBcm^−1^MHz for tissue (Hoskins 1994). To ensure clear images of the bubbles could be obtained the percentage of 10 μm orgasol scatterers was reduced from 0.17% to 0.02%. This diluted suspension generated a Doppler background signal that was approximately 1/100th the strength from blood (Martin et al. 2009b). On exiting the insonation volume the bubbles were imaged through a 4 mm rectangular glass bore (S103; Composite Metal Services Ltd., West Yorkshire, UK) using a high speed, 1200 fps camera (EX-F1 Exilim; Casio Computer Co. Ltd., Tokyo, Japan) and microscope imaging system (Stereo Microscope, RZ Meiji Techno, Somerset, UK).

Gaseous emboli were generated using a custom-built bubble maker modelled on a device previously described by Palanchon et al. (2003a). The holder consists of a main channel (4 mm internal diameter), which is placed in the flow stream and a side arm branching at 26° from the vertical which houses a micropipette (TIPMIX05-10; World Precision Instruments, Sarasota, FL, USA). The micropipette is supplied by an air compressor (Airmaster Tiger 14/60 Compressor, Machine Mart Ltd., Nottingham, UK) and a combination of different flow rates (0.0025–0.0038 ls^−1^), inlet air pressure (0.1–5 bar) and micropipette tip size, (0.5 μm or 1.0 μm) can be adjusted to generate a range of bubble sizes. Smaller bubbles (<1 mm) were generated using lower pressures and flow speeds compared with larger bubbles (>1 mm).

Doppler signals were recorded using a weakly focused 2 MHz transducer (SciMed, Bristol, UK, 3 dB beamwidth of 5.8 mm measured 50 mm from the transducer face) and a noncommercial multigate system (Fan et al. 2006) with high dynamic range to avoid signal saturation. An insonation angle of 30° and a sample volume length of 10 mm were used at a depth of 50 mm to the artificial vessel to mimic the insonation geometry of the middle cerebral artery. Two different set-ups were used where the transducer was either aligned or misaligned with the vessel to see what effect this has on signal intensity. The transducer was aligned when the maximum backscattered signal was observed for the above angle and depth and misaligned when the returned backscatter was less than 50% of the maximum intensity under optimised conditions. This reduction in signal represents a worst case scenario for clinical measurements and was achieved by moving the transducer slightly offset from the centre of the vessel until the intensity reduced by 50%.

**Fig. 4:**
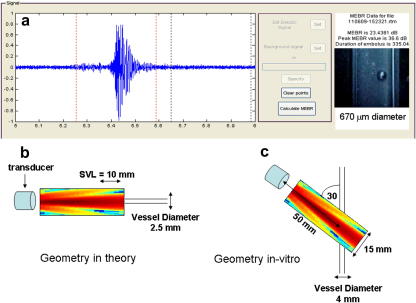
(a) Analysis of embolic signals was performed using a graphical user interface (GUI) programmed in Matlab. A typical Doppler signal from a 670 μm diameter bubble is displayed alongside the recorded image. The embolic signal and background window were manually selected (between the dashed lines) to estimate a peak measured embolus-to-blood ratio (MEBR) in decibels. (b) For the *in vivo* calculation, the beam is aligned with the insonated vessel and a sample volume length (SVL) of 10 mm is assumed. (c) In the *in vitro* experiment, the transducer is aligned at an angle of 30° with the vessel to mimic a more clinically realistic setting. This gives a larger sample volume length of 14 mm. The transducer is placed 50 mm from the centre of the tube.

Raw audio data were extracted and analysed using an in-house program developed in Matlab (Mathworks Inc., Natick, MA, USA) (see Fig. 4). Embolus and background windows were manually selected by the operator to ensure no artefacts were present. The background window was integrated and normalised with respect to time to estimate an average background value, for comparison with the maximum intensity of backscatter from the embolus:

(6) $$MEBR=10\;\log_{10}\left(\frac{I_{E+B}}{I_{B}}\right)\text{dB}$$

where I_E_ is the maximum intensity of the embolic signal and I_B_ is the intensity of the average background audio signal. Note that all MEBR values were converted to EBR values for comparison with the theory.

The recorded videos of the bubbles were examined frame by frame using Quicktime Player (Apple Inc., Cupertino, CA, USA). An image of each bubble was saved as a bitmap image and read into a Matlab GUI developed in-house (see Fig. 4) to measure bubble diameter. The system was calibrated by imaging white, red and black microspheres (Duke Scientific Fremont, CA, USA) of known diameter (1007 ± 20 μm, 500 ± 24 μm and 200 ± 11 μm). The calibrated system is accurate to 40 μm.

For the results shown in Figure 1, the full scattering theory was used to model our specific *in vitro* set-up. In these calculations red-cell aggregates were replaced by 10 μm Orgasol particles and the number density of particles in the solution (“haematocrit”) was altered accordingly. Plasma acoustic properties were replaced with the properties of the BMF. Equation (7) shows how g′ was altered to include these differences for comparison between the approximation and the *in vitro* experiment. See Table 1 for a complete list of values used to calculate g′.

(7) $$g\prime =g-20\;\log_{10}\left(\frac{R}{R_{0}}\right)-10\;\log_{10}\left(\frac{W_{bmf}}{W_{blood}}\right)-10\;\log_{10}\left(\frac{\sigma_{bmf}}{\sigma_{blood}}\right)-10\;\log_{10}\left(\frac{\delta_{bmf}}{\delta_{blood}}\right)$$

## Results

### Testing using a distribution of 10,000 simulated emboli

**Fig. 5:**
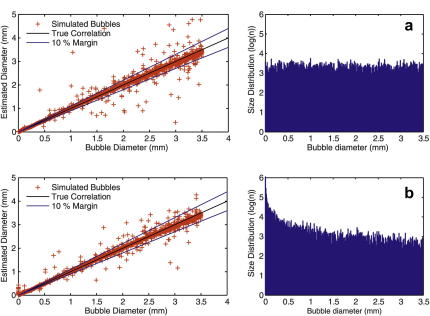
A comparison between bubble diameters for 10,000 simulated emboli randomly generated using (a) a flat and (b) a power law probability distribution (diameters ranging between 1 μm and 3.5 mm) to their estimated diameter using our algorithm. Embolus to background ratio (EBR) values were calculated for the emboli using scattering theory and input into the algorithm to obtain estimated diameters. The outliers result from ignoring the resonant structures on the scattering curve. The histogram in both figures shows the size distribution of bubble diameters on a log scale. If a 10% error margin is assumed, over 97% of the simulated bubbles are within this tolerance for the flat distribution and 91% for the power law distribution. If the bandwidth of the transducer is taken into account, the outliers reduce by ∼ 1%; ignoring the resonant structures using the simplified algorithm will not have a significant impact on accuracy. It should be noted that the inclusion of more 3 μm resonant bubbles in the power law distribution reduces the accuracy of the algorithm slightly (see text for further details).

Figure 5 displays results relating to numerical tests of the scattering theory, showing the distribution of test sizes from 1 μm up to 3.5 mm for both (1) a flat and (2) a power law probability distribution. Ninety-seven percent (97%) of emboli were sized to within 10% of their true size for the flat distribution and 91% for the power law distribution. This drop in accuracy is due to the inclusion of more 3 μm resonant bubbles in the power law distribution. These bubbles may be misclassified as much larger macrobubbles due to their high EBR value (∼50 dB) and it is appropriate to discuss the probability of major oversizing due to these resonant bubbles further.

From Figure 5, for the 10,000 embolus samples simulated, there are a very small number of emboli with an estimated size greater than 1 mm of their true value. These are due to the 3 μm resonant bubbles and those bubbles that lie within a 1 μm range around the resonance. In practical clinical situations, gaseous emboli of this size are very short lived and the power law distribution we have used overestimates the number of gaseous emboli that are likely to fall into this range. To reduce this potential source of error, we recommend that all gaseous emboli recorded with an EBR of less than 30.5 dB are not sized. While this means that no bubble of less than around 18 μm can be sized, it limits the possibility of gross oversizing due to inclusion of those bubbles within a 1 μm range of the resonance. Gaseous emboli of this size are not likely to be clinically significant as they dissolve rapidly (Branger and Eckmann 1999). To demonstrate this, we take simulated data from a steeper power law distribution, which has five times more small gaseous emboli (diameters between 2–4 μm). EBRs are calculated using the full theoretical curve and then the 30.5 dB cutoff is applied. By applying the sizing algorithm on this set, we find that less than 1.3% of emboli with EBRs greater than 30.5 dB are significantly oversized (*i.e*., having estimated diameters greater than 1 mm of their true value).

### *In vitro* testing

**Fig. 6:**
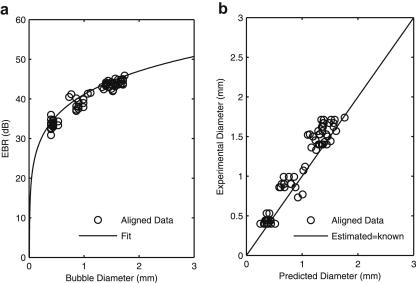
(a) Experimental embolus to background ratio (EBR) values for 72 aligned bubbles and their measured diameters compared with the fitted function. (b) Good agreement is found between predicted diameters and measured diameters for the aligned bubbles using the algorithm.

**Fig. 7:**
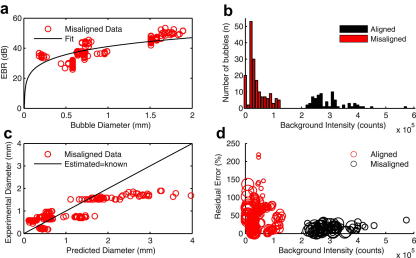
(a) Experimental embolus to background ratio (EBR) values for 175 misaligned bubbles and their measured diameters compared with the fitted function. (b) Histogram of background counts for the aligned and misaligned data. A clear separation in intensity is visible. (c) Large deviations are seen for the predicted bubble diameter compared with the measured diameter for misaligned data, particularly for larger bubbles. (d) The residual error expressed as a percentage for aligned and misaligned bubbles against their background signal intensity. Each marker is proportional to bubble size. Aligned bubbles have on average approximately 15% residual error compared with 49% for misaligned bubbles.

To test whether the algorithm could predict bubble sizes *in vitro,* we obtained Doppler embolic signals from a total of 247 bubbles with diameters ranging between 0.21 and 1.73 mm. The average standard deviation in bubble diameter was ±40 μm and ±1.4 dB for peak MEBR. The actual error in EBR values for individual measurements is more difficult to estimate. Figure 6 shows the individual bubble measurements for 72 bubbles when the probe was aligned with the vessel (tubing). The spread of each cluster of points is consistent with the relative intensity of the Doppler speckle background, which ranges from 3 to 10 dB (Ringelstein et al. 1998). Figure 7 displays the bubble measurements for the misaligned set-up where large deviations from the fitted function are observed. The algorithm predicts the size of over two-thirds (69%) of the aligned bubbles to within 20% of their true value. Misalignment of the beam produced much less reliable estimates for bubble size, however, with only 30% of bubbles correctly sized to within 20% accuracy.

Although good agreement is observed between the measured EBR values and corresponding bubble diameters for the aligned data, these data highlight difficulties in determining bubble size when the probe is misaligned. For larger bubbles where the EBR curve begins to plateau, so as the difference in the theoretical EBR between a 1 mm and 2 mm bubble is less than 5 dB, experimental errors of similar magnitude can propagate as large (50%) errors in sizing large macrobubbles. For example, an error of only 3 dB translates to a 42% error in bubble size for a 1 mm bubble.

## Discussion

A major barrier to implementation of bubble sizing using TCD is the difficulty involved in using the full scattering theory to interpret Doppler embolic signal intensities. The principal aim of this study was to develop a straightforward method for translating embolic signal intensity to prediction of bubble size. We wanted to develop an algorithm that could be implemented in real-time during clinical monitoring and adjusted to reflect patient specific vessel diameter and variations in haematocrit. Comparison of our proposed sizing algorithm with the exact relationship between embolus size and EBR based on solving more complicated equations using scattering theory showed promising results. Tests on simulated data showed that our algorithm is theoretically capable of sizing over 90% of signals within a 10% error margin.

The algorithm can also be adjusted to account for variations in some of the parameters between patients. For example, both vessel radius (R) and haematocrit (H) will affect predicted EBR values. Van der Zwan et al. (1993) measured the variability of the MCA in 23 human brains and found the average diameter to be 2.7 mm (min 2.31 mm, max 3.46 mm). The maximum difference calculated from the scattering model [eqn (1)] using this spread in MCA diameters was 3.5 dB, which will only contribute significant error when sizing macrobubbles.

Haematocrit and red-cell aggregation can also cause significant variations in the blood backscatter coefficient. The haematocrit is proportional to the number density of scattering red cells in the blood and a packing factor *W* incorporates red-cell aggregation. In cardiac surgery, haematocrit drops precipitously during cardio pulmonary bypass (CPB) due to blood loss and blood cell dilution (Vretzakis et al. 2011). Up to ∼5 dB difference was calculated for macrobubbles greater than 1 mm in diameter for H concentrations between 25% and 45%. This could lead to serious undersizing of large bubbles if not taken into account during clinical measurements. Using eqn (3) to incorporate these variables into our algorithm, a more accurate estimation of bubble size can be made for individual patients.

Our *in vitro* experiments show that practical sizing of emboli *in vivo* is likely to be adversely affected by experimental uncertainties and misalignment between the beam and the vessel. The shallow gradient of the EBR vs. bubble radius curve for large emboli means that even small errors in MEBR will propagate into large errors in estimates of bubble size. Our sizing algorithm, therefore, predicts likely bubble size, but errors can be up to 50% for large bubbles >1 mm (although the vast majority of bubbles sized using a well aligned beam were sized to within 20% of the optically measured size).

Incomplete insonation of the vessel is indicated by a reduction in backscatter power of the background signal. In measurements where the vessel was fully insonated 69% of bubbles were sized to within 20% of the more accurate value obtained optically, which reduced to 30% where the backscatter was less than 50% of its maximum due to misalignment. Figure 7d displays the residual error (the difference between the estimated size and measured size divided by the measured size) as a percentage for each sized bubble against the measured background with the markers proportional to bubble size. It can be seen (Fig. 7c) that misalignment of the beam and large errors in MEBR translate to poor bubble sizing. This highlights the necessity for good beam-vessel alignment in a clinical setting. Angell and Evans (2003) showed that by varying the angle of intersection between the vessel and beam, and for different offsets between the vessel and sample volume, the range of possible EBRs was approximately 4 dB with perfect alignment to over 12 dB where the beam was misaligned. The same study also showed that beam distortion through temporal bone affects intensity measurements and can increase relative MEBR by ∼3 to 4 dB. To ensure reproducible information from embolic signals “*every effort must be made to find the strongest signal from blood for each patient”*.

Our *in vitro* experiments demonstrated that emboli sizes can be estimated based on analysis of MEBR values. However, users will need to take care to optimise the quality of data and should be prepared to accept that there may be large errors associated with estimated sizes. Given these limitations, provided that errors are properly considered, the algorithm we have presented could prove to be a useful research tool for sizing bubbles intraoperatively by distinguishing small benign bubbles from clinically significant macrobubbles. Further work testing the algorithm during clinical monitoring is underway.

## Conclusion

In this article, an approach to gaseous embolus sizing using Doppler ultrasound has been demonstrated. A combination of numerical modelling and *in vitro* experiments were used to establish bounds on the accuracy of the method. To help translate embolic signal intensity to gaseous embolus size, a simplified expression for gaseous embolus sizing was introduced. Inversion of this function permits estimation of bubble radius from peak MEBR values. Numerical testing shows that over 90% of emboli can be sized to within 10% of their known values for diameters ranging between 1 μm and 3.5 mm. *In vitro* validation demonstrates that sizing of bubbles is possible using ultrasound but also highlights the importance of considering experimental error. Once measurement errors are introduced large percentage uncertainties in embolus sizing can be seen, particularly for large bubbles where the relationship between EBR and embolus size reaches a plateau. Despite these limitations, we believe that even a crude indication of bubble size will be useful in clinical settings such as cardiac surgery where patients receive a mixture of small and large air bubbles and there are currently no methods to distinguish benign microbubbles (<100 μm) from clinically significant macroemboli.

## Figures and Tables

**Table 1:** Numerical values used in modelling the relationship between embolic signal intensity and air bubble diameter in blood (*in vivo*) and in our laboratory phantom (*in vitro*)

Modelling assumptions *in vivo*		
Blood properties	Density kg·m^−3^	Speed of sound m·s^−1^
Blood	1055	1580
Red cell aggregate	1090	1640
Plasma	1030	1542
Air	0.0011387	353.3
Red cell assumptions	Diameter	2.34 μm
	Number density (δ_blood_)	5 × 10^15^ m^−3^
	Cross-section (σ_blood_)	1.22 × 10^−20^ m^−2^
	Hematocrit (H)	45%
	Packing factor (W)	0.0253
Insonating artery	Vessel radius (R)	1.25 mm
	SVL	10 mm

Modelling assumptions *in vitro*		
BMF properties	Density kg·m^−3^	Speed of sound m·s^−1^
BMF	1037	1547
Orgasol particles	1060	2340
Plasma (glycerol mixture)	1022	1550
Orgasol assumptions	Diameter	10 μm
	Number density (δ_bmf_)	1.14 × 10^13^ m^−3^
	Cross-section (σ_bmf_)	2.49 ×10^−18^ m^−2^
	Hematocrit (H)	0.006%
	Packing factor (W)	1
Insonating artery	Vessel radius (R)	2 mm
	SVL	14 mm

BMF = blood mimicking fluid; SVL = sample volume length.
